# Haematuria as a presentation of metastatic oesophageal carcinoma

**DOI:** 10.1186/1477-7800-2-4

**Published:** 2005-02-20

**Authors:** R Hargunani, S Al-Dujaily, AKS Abdulla, DR Osborne

**Affiliations:** 1 Department of Urology, Basildon University Hospital, Essex, UK

## Abstract

Haematuria is a classical symptom of urological disease often signifying a primary bladder cancer. Rarely, however, the presence of blood in the urine can be due to secondary spread of tumours into the bladder from distant sites. Notably this has been reported to occur in breast cancer, malignant melanoma and gastric cancers. Haematuria due to spread from a primary oesophageal cancer to the bladder has never been reported. We present a case of haematuria confirmed histologically to be due to metastases from a primary oesophageal tumour. Oesophageal cancer is capable of spread to all three neighbouring compartments (abdomen, chest and neck) and therefore has the potential to spread to unusual sites. Clinicians should always carefully regard haematuria in a patient previously treated for cancer and retain a high index of suspicion for distant metastases as being the cause.

## Background

Haematuria is a commonly encountered symptom. It often represents the presence of serious disease such as a malignancy within the bladder. The majority of bladder tumours tend to be primary, and histologically these are usually transitional cell carcinomas. We present a case of haematuria which occurred due to metastases from a primary oesophageal carcinoma diagnosed 2 years prior and treated curatively.

## Case presentation

A 45-year old male presented to our unit with acute onset macroscopic haematuria. His past medical history was significant in that he had been diagnosed with adenocarcinoma of the distal oesophagus 2 years prior and had undergone curative resection after neo-adjuvant chemotherapy. At that time the tumour was found to be poorly differentiated with evidence of local nodal spread. He had been reviewed regularly by the oncologists and remained asymptomatic until the onset of frank haematuria.

**Figure 1:**
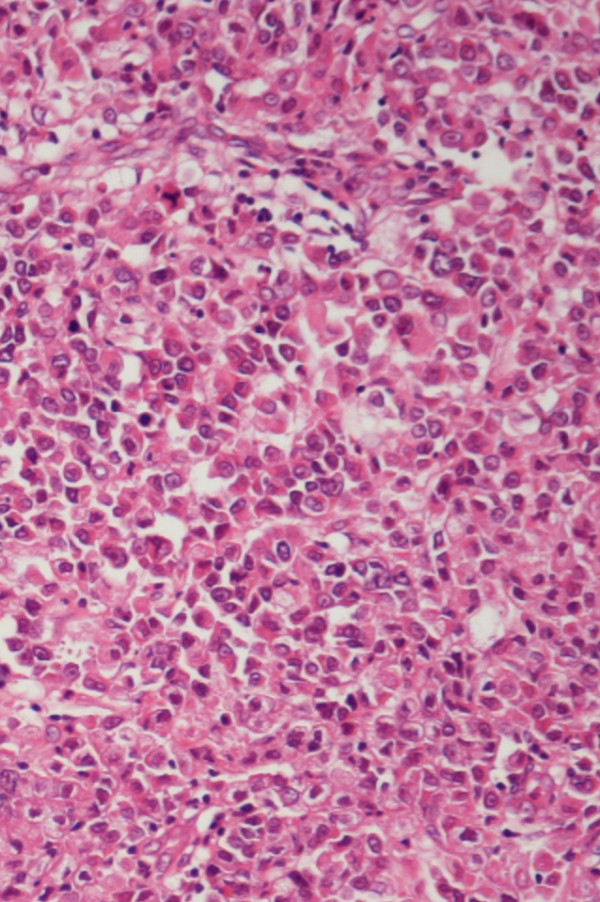
Original oesophageal adenocarcinoma (H & E stain).

**Figure 2:**
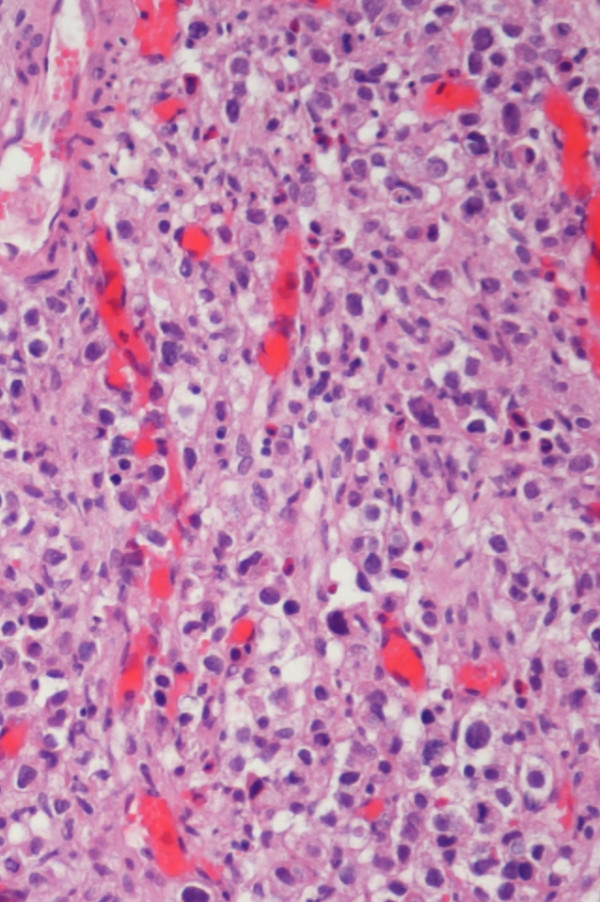
Metastatic tumour to the bladder (H & E stain). Both light micrographs demonstrate extensive infiltration by a poorly differentiated adenocarcinoma with the same histopathological features.

He subsequently underwent cystoscopy, which revealed a solid bladder tumour on the right lateral wall, which was treated with trans-urethral resection. Pathological examination confirmed a poorly differentiated mucus-secreting adenocarcinoma, identical histologically to the original oesophageal tumour (Figures 1 and 2).

A diagnosis of metastatic oesophageal adenocarcinoma to the bladder was made. A CT scan did not demonstrate any pelvic tumour outside the bladder and therefore metastasis by the trans-coelomic route was essentially excluded, indicating haematogenous spread of the primary oesophageal carcinoma. The patient was referred for further oncological therapy but unfortunately died 4 months later from disseminated carcinoma.

## Discussion

Metastatic tumour spread to the bladder constitutes approximately 2% of all bladder neoplasms [1]. Gross haematuria occurs relatively infrequently in secondary tumours of the bladder as most lesions are small and infiltrate the bladder wall without causing ulceration of the mucosa [2]. Therefore most metastases to the bladder remain asymptomatic and often undiagnosed.

The bladder can be the recipient of metastatic tumour spread from a potentially large variety of primary sites. Most commonly direct invasion can occur from adjacent tumours of the lower gastrointestinal tract (33% of secondary neoplasms), prostate (19%) and female genital tract (11%) [1]. Less commonly, distant metastases have been described, notably from the stomach, skin, breast and lung in descending order of frequency [2-5]. The management and prognosis of such tumours can differ significantly from that of primary bladder tumours since they are often indicative of late disease.

Despite curative intent, surgical resection of oesophageal adenocarcinoma is associated with an overall tumour recurrence rate of 66% at 5 years [6]. The lymphatic drainage of the oesophagus is longitudinal via the submucosal plexus and not segmental. As a consequence lymph node metastases can occur relatively early in all three compartments (abdomen, chest and neck) regardless of the location of the primary tumour [7].

In autopsy studies, isolated lymph node metastases were found in approximately one half of patients with end-stage oesophageal carcinoma, with a similar proportion having combined lymph node and visceral metastases. Isolated visceral spread however is rare, accounting for only a handful of cases of primary oesophageal tumour spread [8].

Notable sites of haematogenous dissemination of primary oesophageal carcinoma to distant organs include bone, liver, skin, lungs, adrenals, brain and peritoneum in descending order of frequency [6]. A few authors, most notably in Japan, have described cases of oesophageal cancers metastasising to the kidney. These cases may present with haematuria but often are associated with flank pain [9-14].

Interestingly, rare synchronous primary tumours of the bladder and oesophagus have been described [15,16] but haematuria due to secondary spread of oesophageal cancer has never previously been reported.

## Conclusion

Haematuria may be the only clinically apparent symptom of metastatic tumour spread to the bladder from a potentially large number of primary sites and therefore should be considered by all clinicians irrespective of specialty.

## Competing interests

The author(s) declare that they have no competing interests.
